# Circumferential thoracolumbar corrective fusion with an anterior interbody fresh-frozen femoral head allograft for osteoporotic lower acute kyphosis: a case report

**DOI:** 10.1186/1752-1947-3-137

**Published:** 2009-11-19

**Authors:** Naohisa Miyakoshi, Eiji Abe, Yoichi Shimada

**Affiliations:** 1 Division of Orthopedic Surgery, Department of Neuro and Locomotor Science, Akita University School of Medicine, 1-1-1 Hondo, Akita 010-8543, Japan; 2 Department of Orthopedic Surgery, Akita Kumiai General Hospital, 1-1-1 IIjima-Nishibukuro, Akita 011-0948, Japan

## Abstract

**Introduction:**

Lower acute kyphosis (LAK) is a postural deformity caused by severe osteoporotic vertebral collapse at the thoracolumbar junction. Corrective surgery is indicated for severe cases, but no case report using a fresh-frozen femoral head allograft was found in the English literature.

**Case presentation:**

A 69-year-old Japanese woman with severe LAK with osteoporotic vertebral fractures from T11 to L2 complained of severe back pain and difficulty in walking. The rigid kyphosis measured 74° from T10 to L3. The patient underwent an anterior release and interbody fusion using a fresh-frozen femoral head allograft (T11-L3) and a posterior instrumented fusion (T10-L3). Postoperatively, kyphosis was corrected to 28°, and the patient's symptoms were alleviated. The allograft bone was fully incorporated 1 year postoperatively. A new vertebral fracture at T10 occurred after 2 years, resulting in a slight loss of correction. A kyphosis angle of 35° at 2 years was maintained at 12 years (age, 81 years). She remained free of back pain and able to walk without a cane over the 12-year follow-up.

**Conclusion:**

For treatment of severe osteoporotic LAK, anterior reconstruction is essential to obtain good spinal alignment and prevent recurrence. A fresh-frozen femoral head allograft, in combination with rigid posterior instrumented fixation, fulfills this function.

## Introduction

Lower acute kyphosis, resulting in localized kyphosis of the thoracolumbar junction with compensative thoracic lordosis, is one of the postural deformities caused by severe osteoporotic vertebral fractures at the thoracolumbar junction [1]. Lower acute kyphosis is less common compared with other osteoporotic postural deformities, such as round back and hollow round back, and may be rare in Western countries; however, it is often seen in Asian countries, including Japan. The weakness of lumbar back muscles associated with this deformity leads to additional deterioration [2]. Elderly Asian women who work in forward-bending postures are predisposed to weakness of the lumbar back muscles with degeneration and atrophy [3,4]. This deformity causes severe back pain at the apex of the kyphosis and impairs physical activity and quality of life (QOL) [1]. Corrective surgery is indicated for severe cases, but no case report using a fresh-frozen femoral head allograft was found in the English literature. We report a case showing excellent surgical outcome at the 12-year follow-up after circumferential thoracolumbar fusion with an anterior interbody fresh-frozen femoral head allograft for lower acute kyphosis.

## Case presentation

**Figure 1:**
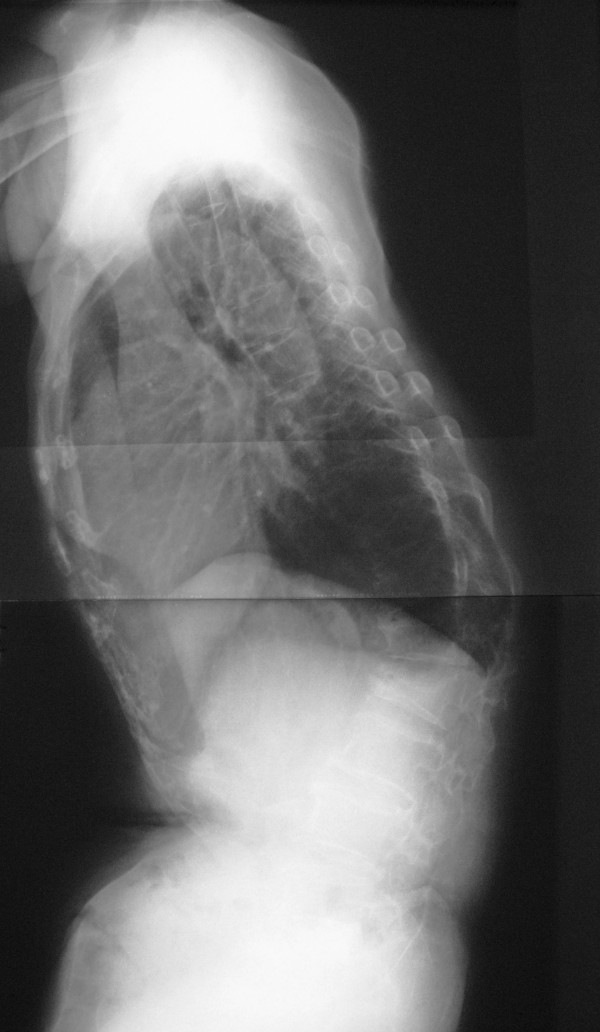
**Preoperative standing lateral radiography of the spine showing osteoporosis and severe lower acute kyphosis (obvious thoracolumbar kyphosis with thoracic lordosis)**.

A 69-year-old Japanese woman with osteoporosis and lower acute kyphosis was referred to our hospital complaining of severe back pain and difficulty in walking. The patient's physical examination demonstrated an obvious thoracolumbar kyphosis with a normal, lower extremity neurologic status. Back pain worsened in intensity and frequency during standing and walking and did not respond to treatment with pharmacotherapy, bracing, or trigger point injections. Protruded spinous processes of the apex with back muscle atrophy resulted in irritated skin, and the patient could not lie on her back. Radiography of total spine showed severe lower acute kyphosis with osteoporotic vertebral fractures of T11, T12, L1, and L2 (Fig. 1). The kyphosis was rigid, measuring 70° from T11 to L2, and correcting to 67° on hyperextension. The kyphosis at the upright position from T10-L3 was 74°.

The patient met the criteria for surgery and underwent an anterior release and interbody fusion followed by a posterior instrumented fusion. A left side extrapleural-retroperitoneal approach with T11 rib resection, anterior spinal release, discectomies, and interbody bone grafting were performed from T11 to L3. For interbody fusion, Harms Cage (DePuy Spine, Raynham, MA, USA) and harvested autograft T11 rib were used for T12-L1 level and 15-mm slices of fresh-frozen femoral head allograft were used for T11-T12, L1-L2, and L2-L3. The fresh-frozen femoral head allograft was obtained from living donors who had undergone total hip arthroplasty. The posterior instrumented spinal fusion using CD System (Medtronic Sofamor Danek, Memphis, TN, USA) was performed from T10 to L3. Partial facetectomies were performed at each level. Transpedicular fixation was obtained bilaterally from T11 to L3. Downgoing transverse process hooks were placed bilaterally at T10, and upgoing laminar hooks were placed bilaterally at L3. Bone chips from the femoral head allograft mixed with resected spinous processes were grafted posteriorly. Postoperatively, kyphosis was corrected to 28°, as measured from T10 to L3. Symptoms were alleviated after surgery.

**Figure 2:**
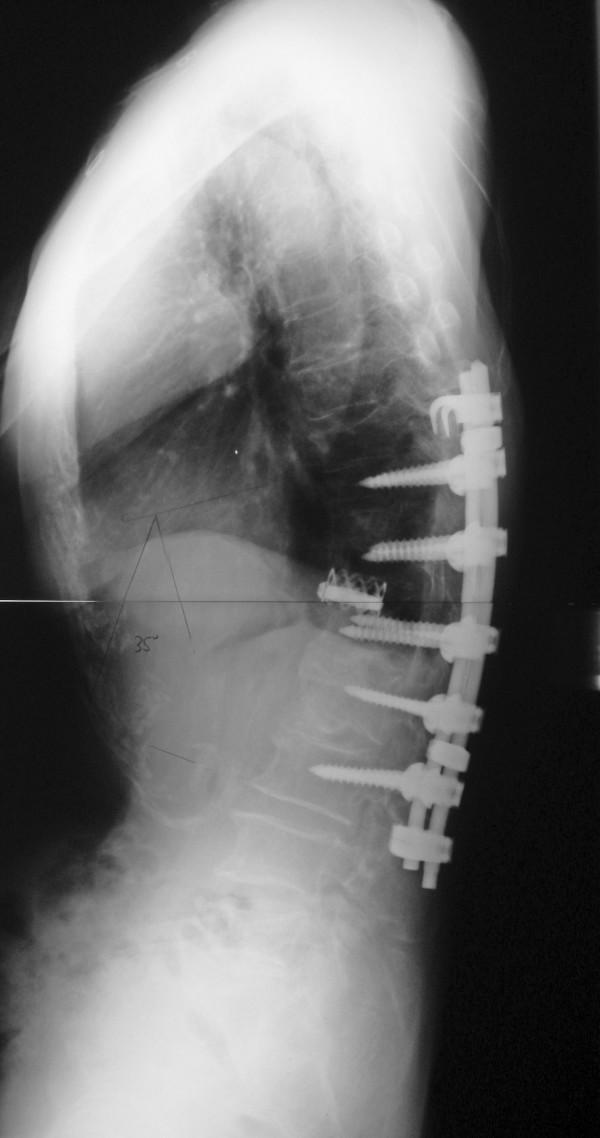
**Standing lateral radiography of the spine obtained 2 years after surgery**. Good correction was obtained.

**Figure 3:**
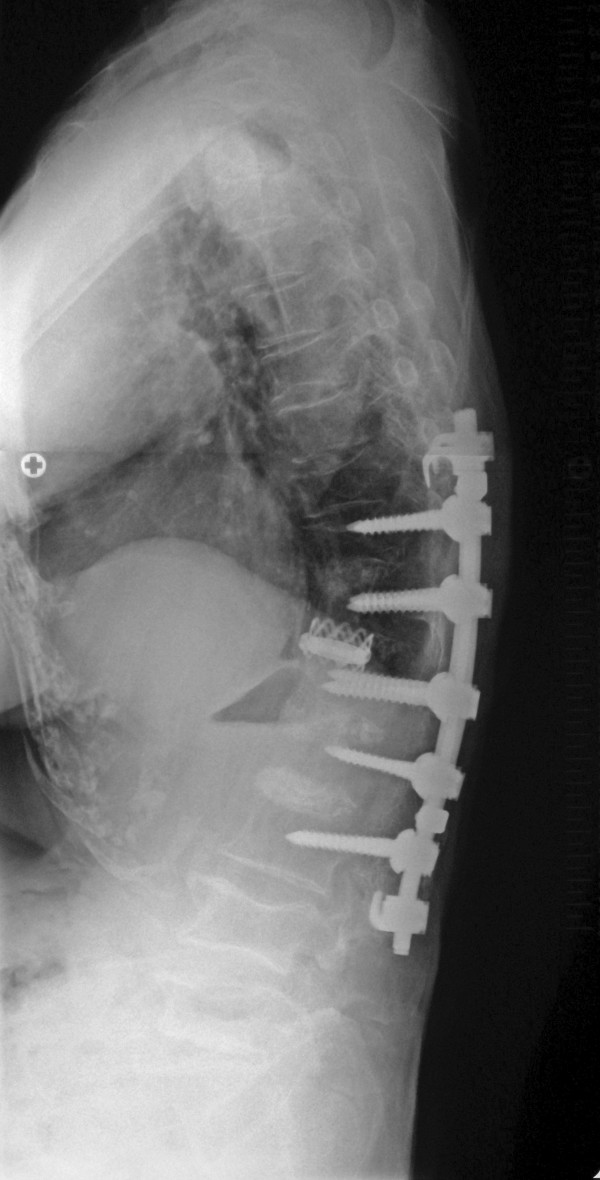
**Standing lateral radiography of the spine obtained 12 years after surgery**. No correction loss was observed up to final follow-up.

A thoracolumbar brace was applied for 6 months. Interbody solid fusion from T11 to L3 with incorporation of the femoral head allograft was seen by 1 year postoperatively. A new vertebral fracture of T10 occurred 2 years postoperatively, resulting in a slight loss of correction (35°, T10-L3). However, no additional vertebral fractures occurred thereafter. After surgery, the patient started osteoporosis pharmacotherapy with alfacalcidol (1 μg/day) for 5 years, followed by alendronate (5 mg/day) or risedronate (2.5 mg/day) until present. Kyphosis corrected to 35° 2 years postoperatively (Fig. 2) was maintained at the patient's 12-year follow-up evaluation (Fig. 3). She remained free of back pain and able to walk without a cane at her 12-year follow-up (age, 81 years).

## Discussion

Severe osteoporotic lower acute kyphosis is a rare spinal disorder that impairs a patient's physical activity and QOL due to intolerable back pain and difficulty in walking. Spinal sagittal malalignment, showing obvious local kyphosis at the thoracolumbar junction (caused by severe vertebral collapse) and back muscle weakness, induce additional fractures, resulting in progressive angular kyphosis. Surgical spinal correction is indicated to reduce the patient's symptoms. In the elderlies, less invasive surgeries such as vertebroplasty and kyphoplasty are generally recommended for osteoporotic spine. However, vertebroplasty or kyphoplasty alone cannot correct the rigid spinal kyphosis. From a mechanical point of view, an anterior-column injury should be approached anteriorly [5]. For the treatment of severe osteoporotic lower acute kyphosis, anterior release and anterior support reconstruction is important to obtain good spinal alignment and prevent recurrence.

A femoral cortical ring allograft (femoral shaft allograft) is commonly used for anterior interbody fusions in Western countries [6,7]. However, the allograft, especially cortical bone of the femoral ring, requires at least 18 months before most cortical bone allografts are fully incorporated into the host bone [6]. In addition, the cortical ring may be mechanically too strong for the osteoporotic vertebrae. The subsidence of an implanted material is generally caused by a loss of strength at the implant-vertebral bone junction [8].

A fresh-frozen femoral head allograft fulfills its desired function as an anterior structural graft in combination with rigid posterior transpedicular fixation, maintaining the disk space height achieved at surgery while allowing remodeling and incorporation into a solid anterior fusion [9]. Advantages of this allograft include initial biomechanical stiffness and correction and maintenance of any deformities. In addition, the increased area of surface contact between the allograft and adjacent vertebral body and the placement of the graft under compression improve stability and allow early axial loading. Bendo et al. [9] evaluated 50 cases that underwent lumbar interbody fusions for non-osteoporotic lumbar degenerative diseases with anterior fresh-frozen femoral head allograft as a structural interbody graft material with a mean follow-up of 28 months. Ninety percent of their cases were one- or two-level fusions. In their series, the average time to anterior radiographic fusion was 6 months (range, 4 to 8 months) without correction loss, and the overall fusion rate was 98% [9]. Compared with autogenous bone graft, the lower incorporation and fusion rates of allografts are well known, but the fusion time of the femoral head allograft [9] is shorter than the femoral cortical ring allograft [6].

In Japan, allograft bone grafting is not popular because allograft bones are not commercially available. However, in our case, we could use a fresh-frozen femoral head allograft from living donors who had undergone total hip arthroplasty and obtained excellent long-term outcome.

## Conclusion

For treatment of severe osteoporotic LAK, anterior reconstruction is essential to obtain good spinal alignment and prevent recurrence. A fresh-frozen femoral head allograft, in combination with rigid posterior instrumented fixation, fulfills this function. Excellent surgical outcome without correction loss was maintained over 12 years in the present case.

## Abbreviations

LAK: lower acute kyphosis.

## Consent

Written informed consent was obtained from the patient for publication of this case report and accompanying images. A copy of the written consent is available for review by the Editor-in-Chief of this journal.

## Competing interests

The authors declare that they have no competing interests.

## Authors' contributions

The authors were involved in the writing of the manuscript and patient clinical care. All authors read and approved the final manuscript.
